# Single-Cell Transcriptome Profiling Simulation Reveals the Impact of Sequencing Parameters and Algorithms on Clustering

**DOI:** 10.3390/life11070716

**Published:** 2021-07-19

**Authors:** Yunhe Liu, Aoshen Wu, Xueqing Peng, Xiaona Liu, Gang Liu, Lei Liu

**Affiliations:** 1 Institute of Biomedical Sciences, Fudan University, Shanghai 200000, China; yunhe_liu15@fudan.edu.cn (Y.L.); aswu16@fudan.edu.cn (A.W.); 18111510058@fudan.edu.cn (X.P.); 16111520003@fudan.edu.cn (X.L.); 2 School of Basic Medical Science, Fudan University, Shanghai 200000, China

**Keywords:** single cell, bioinformatics, simulation, clustering, cell type annotation

## Abstract

Despite the scRNA-seq analytic algorithms developed, their performance for cell clustering cannot be quantified due to the unknown “true” clusters. Referencing the transcriptomic heterogeneity of cell clusters, a “true” mRNA number matrix of cell individuals was defined as ground truth. Based on the matrix and the actual data generation procedure, a simulation program (SSCRNA) for raw data was developed. Subsequently, the consistency between simulated data and real data was evaluated. Furthermore, the impact of sequencing depth and algorithms for analyses on cluster accuracy was quantified. As a result, the simulation result was highly consistent with that of the actual data. Among the clustering algorithms, the Gaussian normalization method was the more recommended. As for the clustering algorithms, the K-means clustering method was more stable than K-means plus Louvain clustering. In conclusion, the scRNA simulation algorithm developed restores the actual data generation process, discovers the impact of parameters on classification, compares the normalization/clustering algorithms, and provides novel insight into scRNA analyses.

## 1. Introduction

Single-cell RNA sequencing technology has developed rapidly in recent years. It has gradually become the preferred sequencing technology for researchers in fields including histological variation [1,2,3] and the tumor immune microenvironment [4,5]. However, there are still some shortcomings in the current analysis workflow. The sequencing depth for a single cell (50,000 [3], limited data allocated to too many cells) is insufficient for transcriptome profiling analysis [6]. Thus, the quantification of sequencing depth and some other parameters on classification accuracy is necessary for scRNA analysis.

Several scRNA-seq analysis algorithms based on reasonable assumptions and models have been proposed in the past few years, including Biscuit, K-means plus Louvain clustering, MNN (matching mutual nearest neighbors), and CCA (canonical correlation analysis) in batch effect removing [7,8,9]. However, a few articles used the same analysis workflow or parameter [1,2,3,4,5,10,11,12], leading to quite different analysis results. In the absence of ground truth, it is hard to determine which is better, and the researchers might choose algorithms subjectively.

Simulation is a frequently used option. Simulation refers to using relevant mathematical models to imitate real processes by computer, which in turn generates simulated data. With the pre-defined ground truth and parameters of the simulator, the influence of parameters can be quantified without systematic errors [13]. Several simulation programs for scRNA-seq court data (refers to the quantification result of the mapped reads) have been released, including SPsimSeq, Splatter, SPARSim, and SymSim [14,15,16,17]. These algorithms are all hypothesis-driven instead of data-proposed models [18,19]. The drawback for these algorithms is that the consistency to actual data is difficult to verify in multiple situations except for the features included in the model hypothesis. It is difficult to use a single mathematical model to fit or formulate single-cell expression profiles that constitute several cell populations whose expression is divergent from each other [20].

To address the problems, the SSCRNA program (https://github.com/liuyunho/SSCRNA-v1.0 (accessed on 9 July 2021)) was developed, following a pre-defined ground truth, to simulate scRNA-seq data (fastq data). The SSCRNA program mimicked the actual sequencing process, including the sequencing library building and sequencing process [21], which enabled flexibility to adjust the parameters that might be introduced in each part of actual sequencing. Additionally, consistent with the actual process, a simulated sequencing library could be used several times for sequencing with different parameters. The reliability of the SSCRNA program was verified by comparing the analysis results of both the actual and the simulated data. Using this tool, the impact of sequencing depth on clustering accuracy was quantified, and the performance of current analysis procedures was also evaluated.

## 2. Materials and Methods

### 2.1. Construction of Ground Truth

From the GPL96 platform (https://www.ncbi.nlm.nih.gov/geo/query/acc.cgi?acc=GPL96 (accessed on 9 July 2021)), we collected 225 samples from 11 datasets (Table S1), and the samples were classified into 11 major categories and 42 sub-categories according to the type of enriched cells. We treated the distribution of genes in individual subcategories (at least 3 samples) as independent normal distributions, while the overall distribution of genes was estimated using the mean and variance of each gene from the collected data. Then, 50 cell samples were sampled for each sub-category, and the final dataset with 2100 cells (50 cell×42 categories=2100) was generated.

### 2.2. Correlation Calculation between Samples of Collected Dataset on GPL96 Platform

Whole genes and 530 hemocyte-specific genes were used to calculate the correlation between collected samples (Table S1). The cor function in R environment was used to calculate Pearson’s correlation coefficient between samples, and the data were scaled before the correlation calculation. 

### 2.3. Differential Analysis for Class-Specific Genes

Limma package was used to calculate the differential expression gene for each cluster in a one-vs.-others way. If the gene is obtained as a differential gene for more than four clusters, the gene is deleted, and the remaining genes were viewed as cluster-specific genes.

### 2.4. Default Procedure for Single Cell Analysis

#### 2.4.1. Raw Data Processing Processes

The putative cell barcode was estimated using the “whitelist” function in UMItools, and the cell barcode and UMI were extracted using the “extract” function. STAR software was used to map the reads to reference genome (GRCH38). The featureCounts software was used to determine the gene number according to the map results (Gencode.v29; https://www.gencodegenes.org/human/ (accessed on 9 July 2021) (Hinxton, UK)). The “count” function in UMItools was used to eliminate the polarization effect during the amplification process and to obtain the final scRNA-seq sparse expression matrix.

#### 2.4.2. Count Data Processing Processes (Default Workflow)

R language was used for the subsequent analysis of the expression matrix. The library.size.normalize function of the phateR package was used to make the global library size normalization. The prcomp function was used to reduce the feature dimension and the top 30 feature vectors were selected. The cells were clustered using the Rphenograph package, which was based on the K-means and Louvain algorithm. TSNE plots were used to display the distribution of cells that was incorporated in the Rtsne package. 

### 2.5. Standardization, Dimension Reduction, and Clustering Methods

All the algorithms were implemented in an R environment. The following is the explanation of the function for each algorithm.

#### 2.5.1. 12 Standardization Methods

(1). Count data: Expression matrix obtained using the “count” function in UMItools; (2). Quantile: normalize.quantiles function in the preprocessCore packages; (3). Scale: scale function; (4). Library size standardization: library.size.normalize function of the phateR package; (5). Log transformation: log10 function; (6). Rank standardization: rank function; (7). TPM standardization: the formula of count to TPM ((TPM_i_ = X_i_/l_i_ ∙ [1/(∑_j_ X_i_/l_i_]) where l represents the transcript length, i represents the gene number, j represents cell number) was used to obtain the TPM matrix; (8). EdgeR standardization: using each cell as a sample, standardized factors were calculated using the calcNormFactors function in the edgeR package, common dispersion was calculated using the estimateCommonDisp function, and intergenic range dispersion was calculated using the estimateTagwiseDisp function. The estimated pseudo counts matrix were multiplied with the standardized factors to obtain the final standardized data; (9). Scran standardization: The SingleCellExperiment function in the scran package was used to convert the expression matrix to SingleCellExperiment objects, and the quickCluster function was used to sub-cluster cells. Then, the computeSumFactors function was used to calculate standardized factors within each subclass, and finally, the normalize function was used to complete the standardization.

#### 2.5.2. Two Dimension-Reduction Methods

(1). PCA (Principal Component Analysis): prcomp function; (2). ICA (Independent Component Analysis): fastICA function in the fastICA package.

#### 2.5.3. Five Clustering Methods

(1). Density cluster: findClusters function in the densityClust package; (2). Hierarchical cluster: hclust function; (3). Som (self-organized map) cluster: som function in the som package; (4). K-means cluster: kmeans function; (5). K-means and Louvain cluster: Rphenograph function in the Rphenograph package.

## 3. Results

### 3.1. SSCRNA—A Simulation Program to Generate scRNA-Seq Data

**Figure 1:**
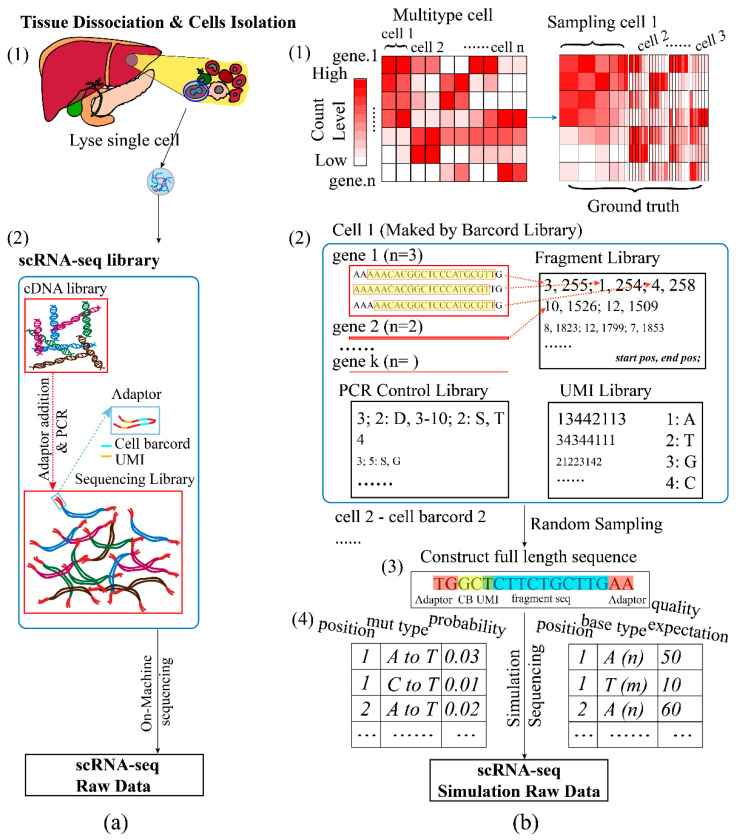
Comparison of the processing of SSCRNA program with the actual process: (**a**) The scRNA-seq process (1. Sample manipulation and cell isolation; 2. Library building: from cDNA to sequencing library). (**b**) The overall process of the SSCRNA program (1. Ground truth: by sampling from the expression data of collected enriched cells; 2. Simulation sequencing library (Consisting of the following four sub-libraries: cell barcode library, gene fragment library, PCR control library, and UMI library); 3. Full-length sequence; 4. Error and quality control files. Example data are available in https://github.com/liuyunho/SSCRNA-v1.0 (accessed on 9 July 2021)).

In the scRNA-seq development, a series of sequencing workflows had arisen, such as SMART-seq2, CELL-seq, and Drop-seq [22,23,24], and the process of all these methods consists of the following three sections: cell isolation and capture, library building, and sequencing (Figure 1a). To generate scRNA-seq simulation data, the SSCRNA program mimics the actual sequencing process.

In actual cell definition (cell isolation and capture), the SSCRNA advised a dataset collected from several previous studies (Methods). Previous count simulators used a mathematical model with parameters (e.g., gamma distribution) to define the state of real cells. Although the use of a parametric model allowed more flexibility in adjusting data shape, they often differed significantly from reality, especially in the case of single-cell data with high resolution. The collection and integration of a large amount of real data avoided the difficulty of estimating the signal-to-noise ratio of simulated data and could fit the real situation more closely. In the dataset, the collected samples were classified into 11 major categories and 42 sub-categories (Table S1), which was approaching the number of the clusters of the current scRNA-seq analysis result. The Pearson correlation between samples among the inter and inner group was verified (shown in Figure S1). The ground truth was sampled from the collected dataset (Methods; Figure 1(b1)).

**Figure 2:**
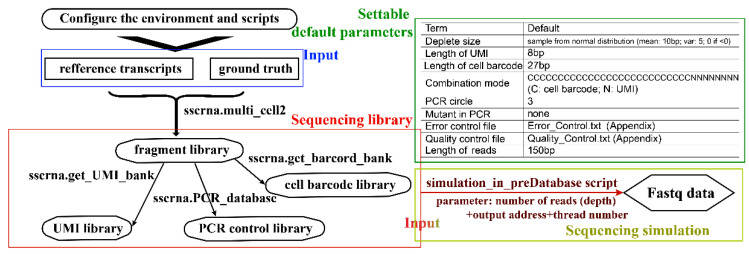
Flow chart and illustration of SSCRNA program and settable parameters.

In the library building simulation, the SSCRNA program implemented a tag-based quantification method, which incorporated UMI (Unique molecular identifier) technology [25] to eliminate the polarization power of amplification. For this implementation, the simulated sequencing library consisted of the following four constituent parts: cell barcode library, gene fragment library, UMI library, and PCR control library (Figure 1(b2)). The reference transcripts sequences (GRCH38) and gene count (Ground truth) were served as input for fragment library simulation (By multi_cell2 function, Figure 2). After randomly missing some sequences from head and tail with a certain probability for a single copy of each gene, the fragment was recorded by the start and end positions relative to the corresponding reference sequence of the gene. All the copies of the genes in one cell were processed in this way to form a single fragment file. The fragment files of all the cells constituted the full fragment library. The UMIs of a certain length matching with each fragment were generated randomly (using the get_UMI_bank function). The cell barcodes corresponding to each cell were generated randomly with a settable hamming distance between them (using the get_barcord_bank function; two hamming distances as the default). The PCR (Polymerase Chain Reaction) simulation emulated the actual exponential amplification process. After a set number of cycles (three was set as default), a PCR control library was produced to record the number of each fragment and potential mutation introduced in the PCR process (using the PCR_database function). Then, the completed sequencing library was fed into the next on-machine sequencing simulation program (Figure 2).

The sequencing simulation could be executed after a set sequencing depth and threads number (using the simulation_in_preDatabase script). The sequenced genes were randomly selected, and their respective components were extracted from four sub-sequencing libraries to form a full-length sequence (based on combination rules of different platforms; Figure 1(b3)). In reads file generating, the sequencing error and base quality were introduced based on the assumption that the error probability and quality expectation for each base rely on the base type, the mutation type, and the position in the sequence (controlled by Error_Profile and Quality_Profile files, Figure 1(b4)). The resulting sequences and corresponding quality were organized into raw seq-data files at last.

The sequencing library produced using SSCRNA was written into the hard disk, which could be used for several sequencing simulations with different parameters for comparison. As in the actual situation, after the scRNA-seq library was built, the on-machine sequencing could be conducted several times. The program incorporated multi-threads to enable fast random search and extract sequences in large sequencing library files and quickly produce massive scRNA-seq simulation data. The implementation of this program provided a framework that considered each part in the actual sequencing process, which could be updated further by adding different parameter sets and models into the relevant part.

### 3.2. The Validation of SSCRNA by Comparing with Real Data

**Figure 3:**
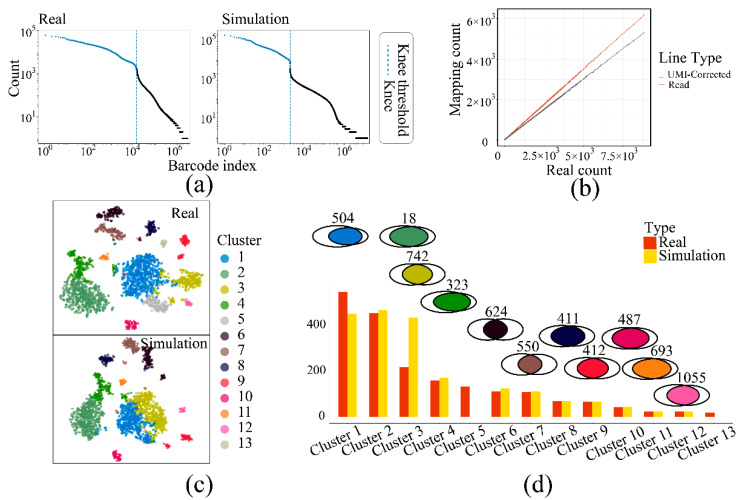
Comparison between simulation data and real data: (**a**) The cell barcode-count curve (left panel: real data; right panel: simulation data). (**b**) The plot of mapping count versus real count with and without correcting using UMI technology. (**c**) The cell distribution (TSNE plot) comparison between simulation and real data (upper panel: real data; lower panel: simulation data). (**d**) The comparison of cell number and cluster-specific genes between corresponding clusters of simulation/real. (Boxplot: cell number; Venn plot: the specific genes. The color of the intersection is the same as (**c**)).

The scRNA-seq data, which assigning limited reads to a large number of cells according to cell barcode [26], gives it a low sequencing depth for individual cells and a high dropout value for the entire expression matrix. To validate the reproducible ability of the SSCRNA program, actual scRNA-seq data (DA1, Table S2) was employed as ground truth for the program’s input to simulate the sequencing data (Table S3) with a similar data size. The features were compared (Table 1; Figure 3a), and the analysis result consistency (Figure 3c,d) was evaluated between the simulated and the actual data.

The corrective effect [27,28] using UMI was verified in the simulation data (Figure 3b), satisfying the actual exponential amplification model, which causes more divergency under more substrates. The cell cluster distribution between the actual and the simulated data was comparable (Figure 3c; Data process pipeline and clustering workflow: UMItools for cells and reads identification, STAR for transcripts mapping, library size factor for normalization [29], prcomp function for dimension reduction and Rphenograph for clustering [30,31]). Cluster-specific genes were identified in both data sets, which exhibited a high degree of intersection (Figure 3d), indicating that the simulated data had a high recurrence rate. Taken together, these results indicated that the SSCRNA program reproduced the actual data and encapsulated the feature of scRNA-seq data.

### 3.3. Applications of SSCRNA to Test the Impact of Sequencing Parameters and Algorithms on Clustering

#### 3.3.1. Impact of Sequence Depth

**Figure 4:**
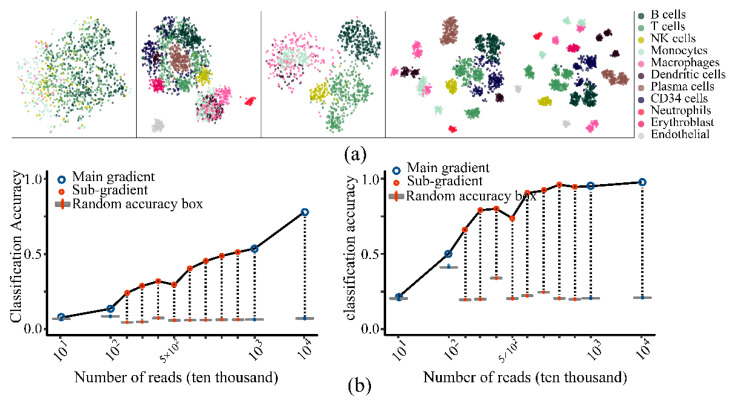
The impact of sequencing depth on clustering. (**a**) The cell distribution plot of simulation data labeled by major category items (simulation data from left to right: RDc.2; RDc.2.1; RDc.2.3; RDc.2.5; RDc.3 in Table S4). (**b**) The classification accuracy curve (left panel: the accuracy of major category; right panel: the accuracy of sub-category. Sub-gradients were set within an interval of the main gradient, where the current real data was located, to make the analysis more precise around the real situation; random accuracy referred to the accuracy obtained by randomly disrupting the cluster index of the analysis result).

Different sizes of sequencing depth were set to simulate data (Table S4; four main gradients; eight8 sub-gradients). As the depth deepens, the actual labels were gradually clustered into blocks in the TSNE plots (Figure 4a; Figure S2B,C). After the classification analysis of each gradient data, the clusters were annotated by the type of true cells that occupy the largest proportion of it. The major category accuracy quickly entered the plateau period, while that of sub-category accuracy is significantly lower, even when the reads per cell reached 17,324 and genes per cell reached 3906 (Figure 4b). Although the cluster number (~40) is close to the current analysis, the average reads and genes detected were much higher than the current sequencing depth (reads per cell around 2000, genes per cell around 600). As a conclusion of this part, the depth of the current actual sequencing data could effectively distinguish main categories, while it was far from being able to distinguish sub-categories under the current analysis workflow.

#### 3.3.2. Reasonableness of Analysis Results in Low Depth

**Figure 5:**
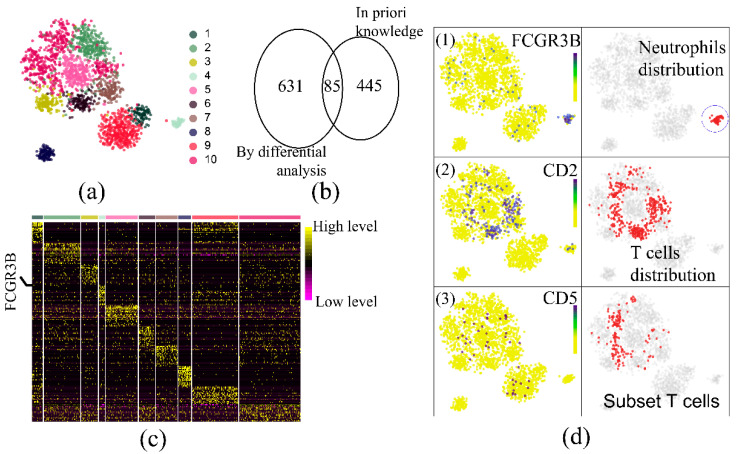
Observations of analysis results at low depth situation. (**a**) TSNE plot of RDc.2.1 data (Table S4); (**b**) Venn diagram of the intersection of cluster specific-genes by differential analysis and the specific genes of prior knowledge; (**c**) Heatmap of cluster-specific genes using differential analysis; (**d**) Cell and gene abundance distribution map (1. FCGR3B gene abundance and neutrophils distribution; 2. CD2 gene abundance and T cell distribution; 3. CD5 gene abundance and subset of T cell (activated memory T cells, Tregs, and Teffs) distribution).

To explore the reasonableness of observations from downstream analysis results under lower sequencing depths, a simulation datum with low accuracy (RDc.2.1, Table S4: Accuracy of major category: 0.6606445; Accuracy of sub-category: 0.2412109), while showing an acceptable cluster distribution (Figure 4a; Figure 5a), was chosen for downstream analysis (1-VS-others differential analysis by limma [32]). The top 20 specific genes of each cluster showed great discriminatory power (Figure 5c). However, only 85 genes of the specific genes (716) overlapped with the hemocyte-specific genes [33] (Figure S3), which meant that the analysis results only recovered less than a quarter of the actual prior knowledge (Figure 5b). FCGR3B (Neutrophils specific gene) was highly expressed in cluster four (Figure 5b), which was consistent with the distribution of neutrophils (Figure 5(d1)). CD2 (T cell-specific gene) was not identified, while its expression was highly compatible with the T cells distribution (Figure 5(d2)), which may result from the nonlinearity distribution of the actual cluster. More surprisingly, at lower depths, the CD5 expression profile did not coincide with the distribution of specific cells that were showing high CD5 expression in prior knowledge (Figure S3; Three sub-categories of T cells: activated memory T cells, Tregs, and Teffs). Therefore, the finding showed that the analysis results of scRNA-seq might not fully reproduce prior knowledge under a low sequencing depth.

#### 3.3.3. Impact of Normalization Algorithms

**Figure 6:**
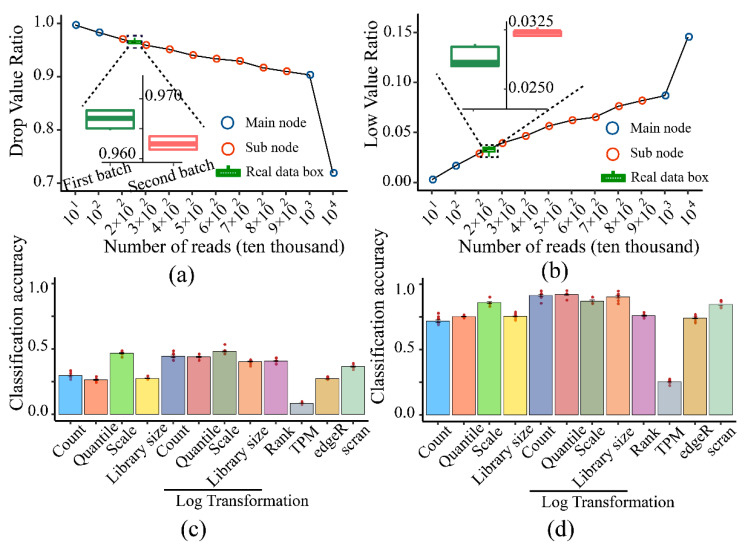
The impact of normalization: (**a**) Relationship between dropout ratio and sequencing depth. (**b**) Relationship between low count ratio and sequencing depth. (Bluepoint: main gradient; redpoint: sub-gradient. Boxplot referred to the two batches of real data (Table S2), and the sequencing depth of the data of second batch (red box) was higher than the first batch (green box)). (**c**) Major category classification accuracies of 12 normalization methods. (**d**) Sub-category classification accuracies of 12 normalization methods.

Considering that the dropout and low count ratios of the expression matrix [20] represented the sparsity of the features that were determinant for classification, a simulated dataset (Table S5) that was consistent with the actual data (Table S2) in these two characteristics (Figure 6a,b)) was chosen to test 12 normalization methods. Different normalization algorithms had quite a considerable impact on clustering accuracy (Figure 6c,d). The TPM and edgeR [34,35], recommended in bulk-RNA seq analysis, performed the worst. Scran [36,37], which can normalize sub-clusters separately, did not perform better. In contrast, a simple z-score normalization method (Scale) contributed the most to classification accuracy. Specifically, log transformation improved the accuracy of other algorithms. Since log transformation and scale normalization were both Gaussian standardization methods, thus, Gaussian standardization was recommended.

#### 3.3.4. Impact of Dimension Reduction and Clustering Algorithms

**Figure 7:**
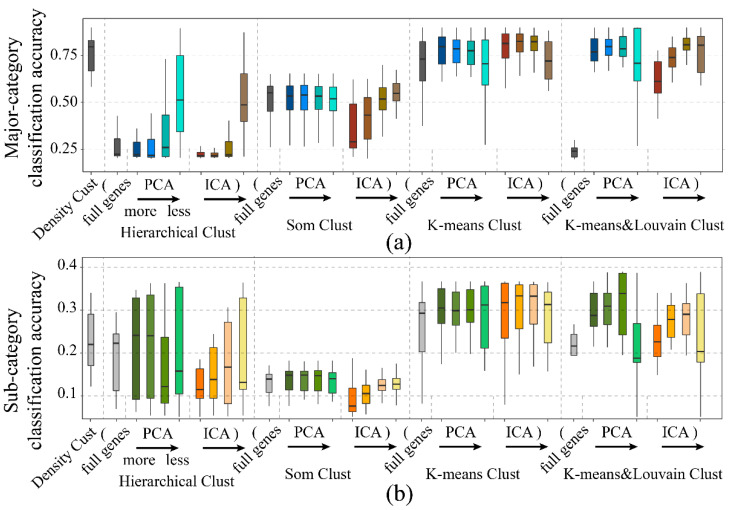
Boxplot of classification accuracy with different combinations of dimension reduction method and clustering method (Dimension reduction: PCA, ICA [38]. Clustering: density cluster [39,40], hierarchical cluster, self-organized map (SOM) [41], K-means, and K-means and Louvain [30]. For each clustering method, the inputs were, from left to right, all genes, the first 100, 70, 40, and 10 features of PCA reduction, the first 100, 70, 40, and 10 features after ICA reduction): (**a**). major category classification accuracy; (**b**). sub-category classification accuracy.

To comprehensively investigate the dimension reduction and clustering algorithms’ performance, the full normalized data of the last result was enrolled in this part. As a result (Figure 7, Figure S4), som and hierarchical clustering were the least effective. The K-means and K-means and Louvain algorithm were outperformed by the others, while K-means was more stable with different feature inputs.

**Figure 8:**
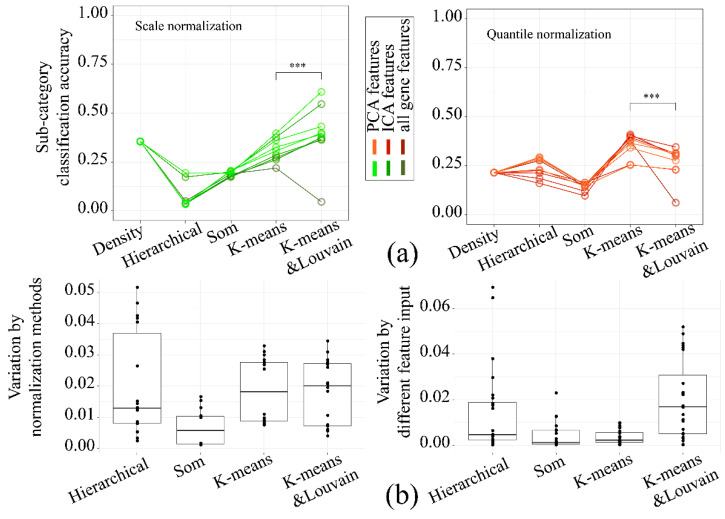
The preference standardized algorithm for different clustering methods and the stability of clustering methods: (**a**) The line plot of classification accuracy (left panel: sub-category classification accuracy of scale normalized data; right panel: sub-category classification accuracy of quantile normalized data; The color of the lines referred to different normalization methods and different input dimension; *** meant the difference was significant at the 0.001 level). (**b**) The boxplot of classification accuracy variance with different clustering method (left panel: variance calculated using different normalization methods; right panel: variance calculated using different dimension reduction methods).

A different combination of normalization and clustering algorithms significantly impacted the overall clustering accuracy. The K-means and Louvain algorithm performed better with scaled data (Figure 8a left panel), while quantile normalized data were more suitable for K-means (Figure 8a right panel). The stability also differed between algorithms (Figure 8b; Left panel: by different clustering features; Right panel: by different normalization method). K-means and Louvain was the most unstable under different feature dimensions as input, which was precisely the opposite of the K-means. In summary, the performance of the five clustering algorithms was somewhat divergent, while K-means and Louvain and K-means should be the best choice under the current workflow, and K-means was a more prudent option.

## 4. Discussion

This project proposed a program (SSCRNA), which utilized a pre-defined ground truth to simulate the scRNA-seq raw data. SSCRNA mimicked the actual sequencing process at all stages, allowing for the generation of raw data according to the parameters of different sequencing platforms. The comparison of the simulation data with the actual data verified the reliability of the program. A ground truth obtained by augmenting the collected data was employed for simulation. We used the simulated data to examine the effect of sequencing depth and analysis workflow on classification accuracy. The test result of sequencing depth suggested that the actual data (10,000 cells) needed at least 50 million reads to achieve better classification results (the classification accuracy of 7 major categories is close to 1, and that of 42 sub-categories is more than 0.5). The test result of the analysis workflow suggested that Gaussian normalization was suitable for the current workflow and K-means clustering was more stable than K-means and Louvain clustering. The scope of the conclusion was limited to the cluster–annotation way. For some other annotation methods that may emerge in the future, it is unknown which normalization algorithm will perform better because it is believed that a minor deformation for the raw data that retains more information might enable a higher potential for upper limits on classification accuracy.

For the fitting of single-cell data properties, researchers have developed several simulation algorithms, including splatter, SPsimSeq, SPARSim, and SymSim, all count simulators [14,15,16,17]. The splat algorithm was recommended in the splatter package, which also inherited a variety of simple algorithms, such as lun2, scDD, etc. [42,43,44,45]. The splat algorithm assumed that the gene expression profile is based on a negative binomial distribution and estimated outlier probability, library size, and dropout indicator from the actual data to generate the observed count as the simulation data. SimSeq was a non-parameter method, which made simulations by sampling from the actual data. Based on this, SPsimSeq was aroused as a semi-parameter method, which made use of Gaussian-copulas to retain the between-genes correlation structure. The SymSim method assumed that the individual gene’s expression follows the stationary distribution of the two-state kinetic model, which used the following three parameters: expression on, off probability, and transcription rate, and specifies the cell state using EVF (extrinsic variability factors). SPARSim, on the other hand, constructed a single-cell count matrix model with a gamma-multifactor hypergeometric distribution model. The common denominator of these methods was that they assumed single-cell expression data satisfy numerous statistical models and estimated the probability distribution of genes through several parameters from actual data, and randomly sampled from this distribution to generate simulation data.

However, for the data with abundant mixed types, which may distribute differently, the algorithm with a relatively simple statistical model and a small size of parameters is unlikely to simulate accurately. First, due to the lack of single population expression data, these algorithms cannot accurately estimate model parameters. In our project, single population expression data were collected in large quantities, and single genes were amplified individually, significantly maintaining the properties of single-cell expression data. Second, the evaluation of the previous method was limited to the comparison of the parameters estimated from the overall distribution and lacked the interpretation of cell population characteristics. Here is an extreme example that swapping data positions randomly in the count matrix will not change the distribution of various parameters (sparsity, coefficient of variation, etc.); the count matrix after the swapping is not consistent with the original matrix. Moreover, although the data processing of scRNA sequencing was analogous to bulk-RNA sequencing data, many parameters, such as sequencing error, mapping efficiency, and sequencing depth, might affect the analysis results at a different level. The previous algorithm ignores the mapping process of reads to count value, while the SSCRNA program allows complete integration of the whole process.

The quantification of the “true state” of the cell population used in the construction of ground truth was derived from the dataset of the array platform. There was a certain degree of subjectivity, such as a quantitative relationship between the signal intensity of the probe and the actual mRNA number. The diversity of gene sequences also introduces noises in the actual sequencing process, which causes lower mapping accuracy. However, this bias was not implemented in the SSCRNA simulator. This study mainly discusses sequencing depth in scRNA-seq analysis, which should be the most apparent parameter on accuracy. Other parameters in the sequencing and analysis process need further exploration, such as the error propensity of different sequencing platforms, different cell barcode estimation algorithms, and the types of errors introduced by different library building processes. 

Several potential analysis directions can be pursued in further analysis, such as exploring the patterns of single-cell expression, screening for new methods for single-cell analysis, and testing the effectiveness of differentiation-related algorithms [46,47]. It is necessary to screen for more relevant algorithms that may generate better results, and the SSCRNA program makes the screening process possible.

## Figures and Tables

**Table 1:** Comparison of characteristics between simulation data and real data.

Data Type	Real Data	Simulation Data
File Size (Fastq)	83.75 G (one of paired files)	87.91 G (one of paired files)
Gene detected in each cell	696.634 (673.759, 719.509)	633.3647(612.8463, 653.8831)
Dropout ratio of full matrix	97.28%	95.38%

## Data Availability

Simulation data were created and analyzed in this study. This data can be found here: (https://github.com/liuyunho/SSCRNA-v1.0 accessed on 8 June 2021).

## Supplementary Materials

The following are available online at https://www.mdpi.com/article/10.3390/life11070716/s1, Figure S1: Correlation heatmap of combined data. Figure S2: Cell distribution of simulation data marked by cluster index, sub-category labels, and major category labels respectively. (A) Cell la-beled by cluster index. (B) Cell labeled by major category labels. (C) Cell labeled by sub-category labels. (Simulation data from left to right: RDc.1; RDc.2.2; RDc.2.4; RDc.2.6; RDc.2.7; RDc.2.8; RDc.4; Table S4); Figure S3: Heatmap of hemocyte-specific genes of combined data (Table S1; CD2, CD5, FCGR3B specified the row in which the corresponding gene was located; Figure S4: Classification accuracy of different clustering methods under different dimension reduction and normalization methods. (A) Classification accuracy without gene feature dimension reduction. (1. Accuracy for sub-category; 2. accuracy for major-category) (B) Sub-category classification accuracy with ICA reduction. (C) Sub-category classification accuracy with PCA reduction. (D) Ma-jor-category classification accuracy with ICA reduction. (E) Major-category classification accuracy with PCA reduction. (the column of picture labelled by (1): 10 features; the column of picture la-belled by (2): 40 features; the column of picture labelled by (3): 70 features; the column of picture labelled by (4): 100 features); Table S1: The information of real data sequencing files; Table S2: The information of read data re-sequencing simulation data; Table S3: the information of collected data from GPL96 platform for augmentation-formed ground truth.; Table S4: The information of simulation data files of augmentation-formed ground truth.;Table S5: The information of simula-tion data files analogous to real data.
