# Supplementary material for: Single-Cell Transcriptome Profiling Simulation Reveals the Impact of Sequencing Parameters and Algorithms on Clustering
Source: Life (Basel). 2021 Jul 19;11(7):716. doi: 10.3390/life11070716 (PMC8304014; doi:10.3390/life11070716)
Supplement: Supplementary file 1 [file life-11-00716-s001.zip › life-1272462-supplementary.pdf]

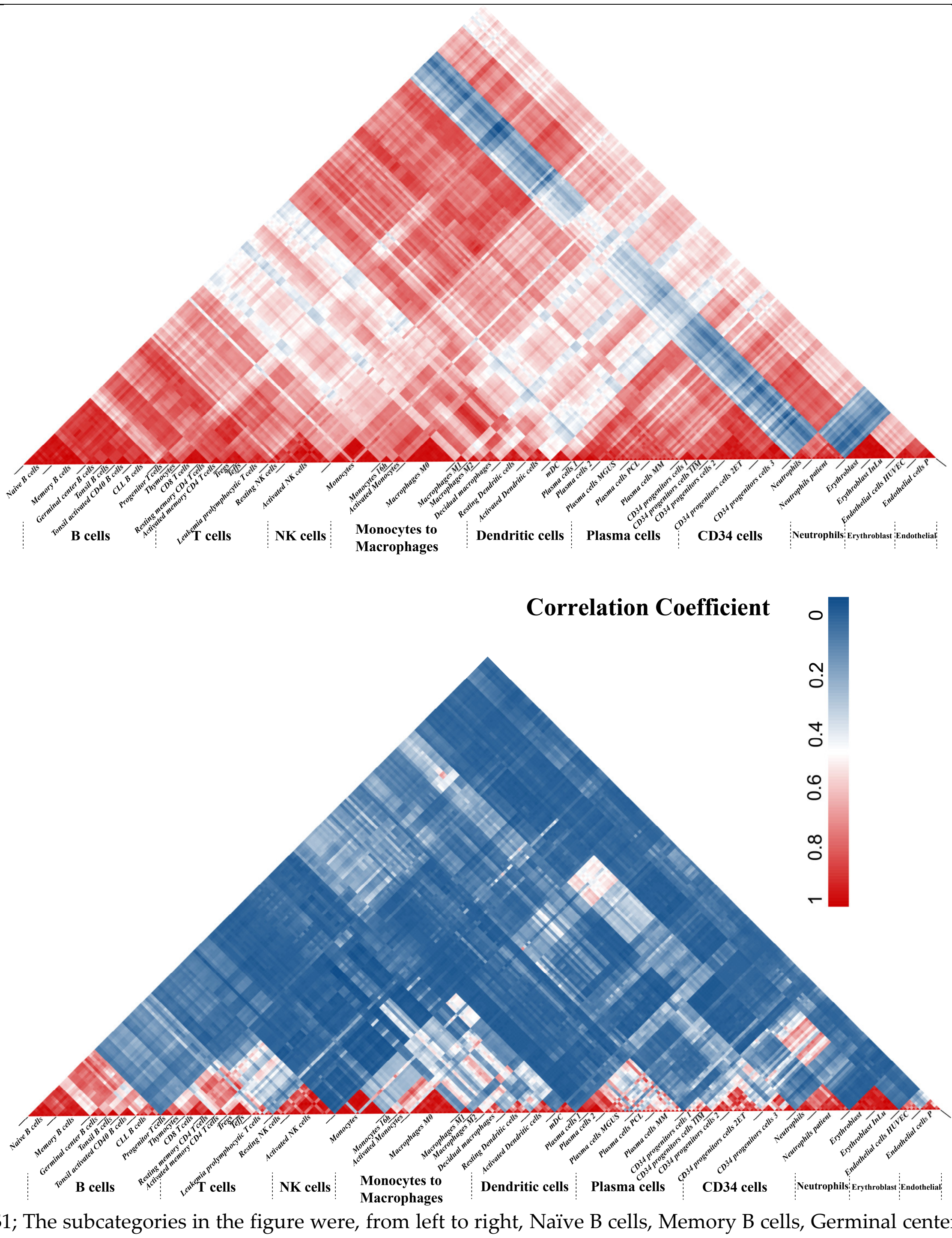

(Table S1; The subcategories in the figure were, from left to right, Naïve B cells, Memory B cells, Germinal center B cells, Tonsil B cells, Tonsil activated CD40 B cells, CLL B cells, Progenitor T cells, Thymocytes, CD T cells, Resting memory CD4 T cells, Activated memory CD4 T cells, Tregs, Teffs, Leukemia prolymphocytic T cells, Resting NK cells, Monocytes, Monocytes 16h, Activated Monocytes, Macrophages M0, Macrophages M1, Macrophages M2, Decidual macrophages, Resting dendritic cells, Activated dendritic cells, mDC, Plasma cells 1, Plasma cells 2, Plasma cells MGUS, Plasma cells PCL, Plasma cells MM, CD34 progenitor cells 1, CD34 progenitor cells 1IM, CD34 progenitor cells 2, CD34 progenitor cells 2ET, CD34 progenitor cells 3, Neutrophils, Neutrophils patient, Erythroblast, Erythroblast InLu, Endothelia cells HUVEC, Endothelia cells P). (upper panel: using whole genes to calculate the correlation; lower panel: using 530 hemocyte-specific genes)

Figure S1. Correlation heatmap of combined data. The dataset containing 11 major categories and 42 sub-categories of immune cell individuals

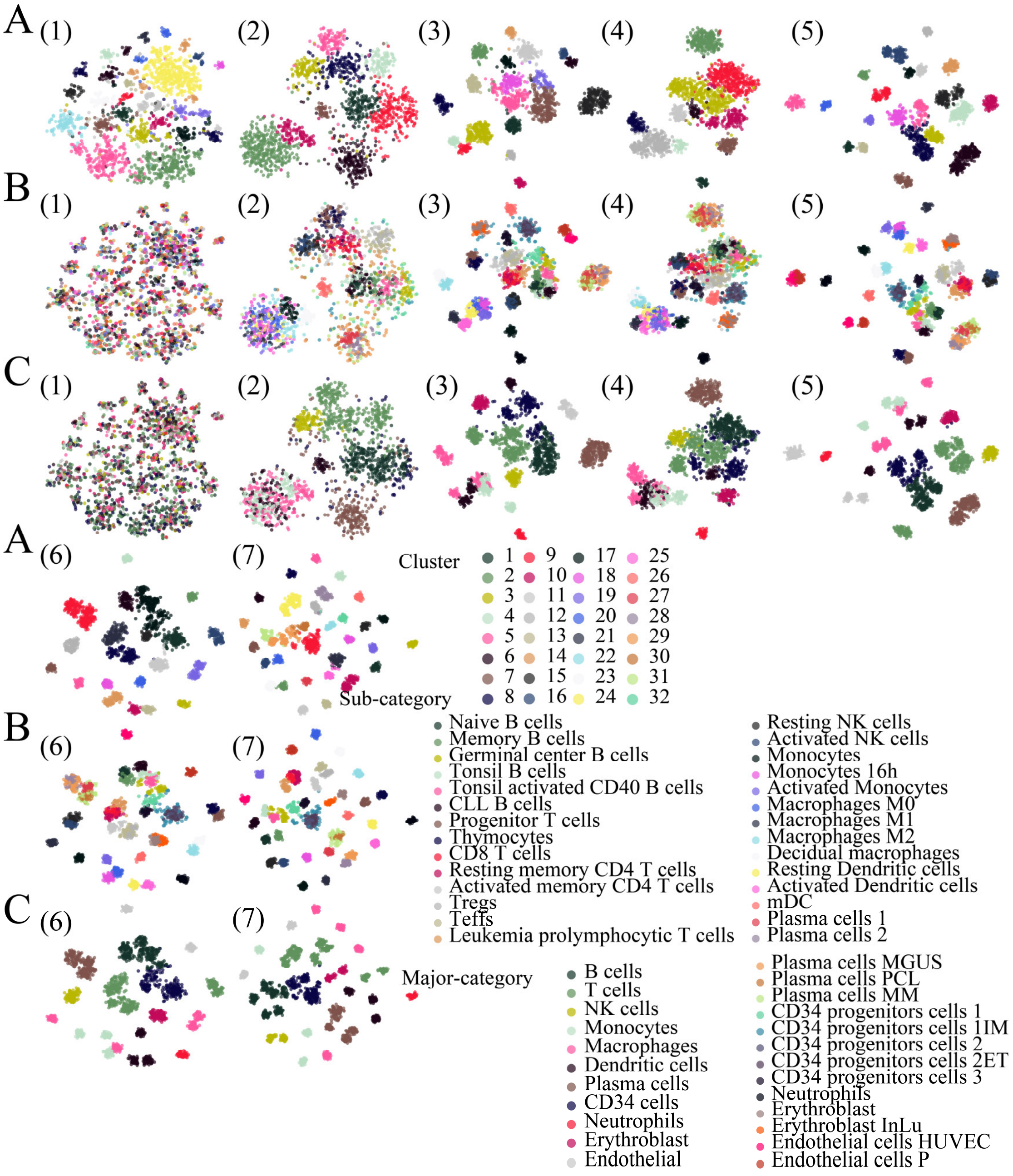

Figure S2. Cell distribution of simulation data marked by cluster index, sub-category labels, and major category labels respectively. (**A**) Cell labeled by cluster index. (**B**) Cell labeled by major category labels. (**C**) Cell labeled by sub-category labels. (Simulation data from left to right: RDc.1; RDc.2.2; RDc.2.4; RDc.2.6; RDc.2.7; RDc.2.8; RDc.4; Table S4)

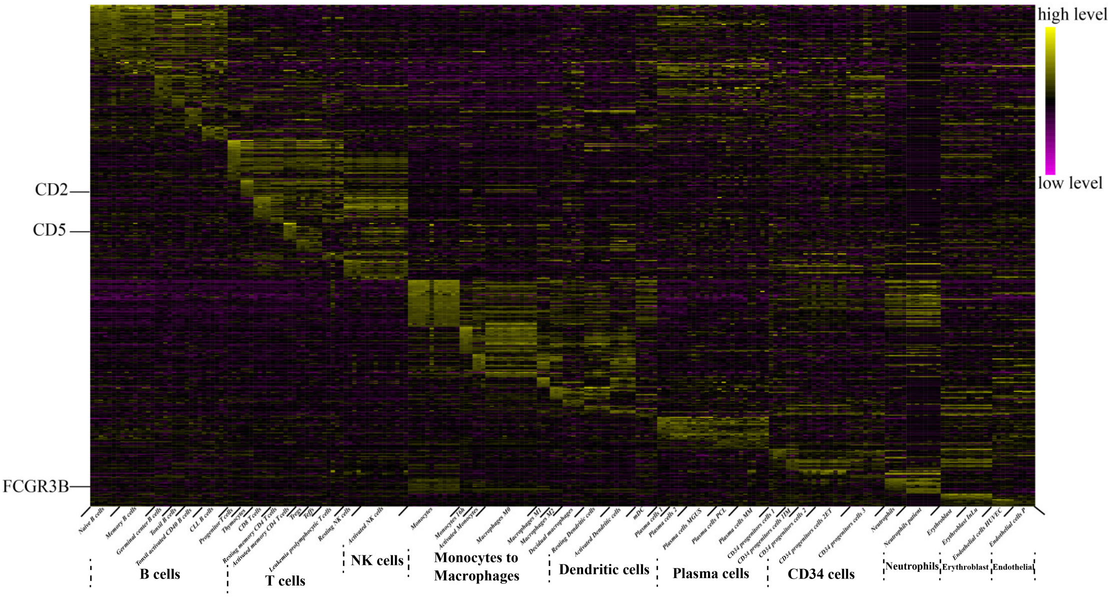

The subcategories in the figure were, from left to right, Naïve B cells, Memory B cells, Germinal center B cells, Tonsil B cells, Tonsil activated CD40 B cells, CLL B cells, Progenitor T cells, Thymocytes, CD T cells, Resting memory CD4 T cells, Activated memory CD4 T cells, Tregs, Teffs, Leukemia prolymphocytic T cells, Resting NK cells, Monocytes, Monocytes 16h, Activated Monocytes, Macrophages M0, Macrophages M1, Macrophages M2, Decidual macrophages, Resting dendritic cells, Activated dendritic cells, mDC, Plasma cells 1, Plasma cells 2, Plasma cells MGUS, Plasma cells PCL, Plasma cells MM, CD34 progenitor cells 1, CD34 progenitor cells 1IM, CD34 progenitor cells 2, CD34 progenitor cells 2ET, CD34 progenitor cells 3, Neutrophils, Neutrophils patient, Erythroblast, Erythroblast InLu, Endothelia cells HUVEC, Endothelia cells P;).

Figure S3. Heatmap of hemocyte-specific genes of combined data (Table S1; CD2, CD5, FCGR3B specified the row in which the corresponding gene was located;

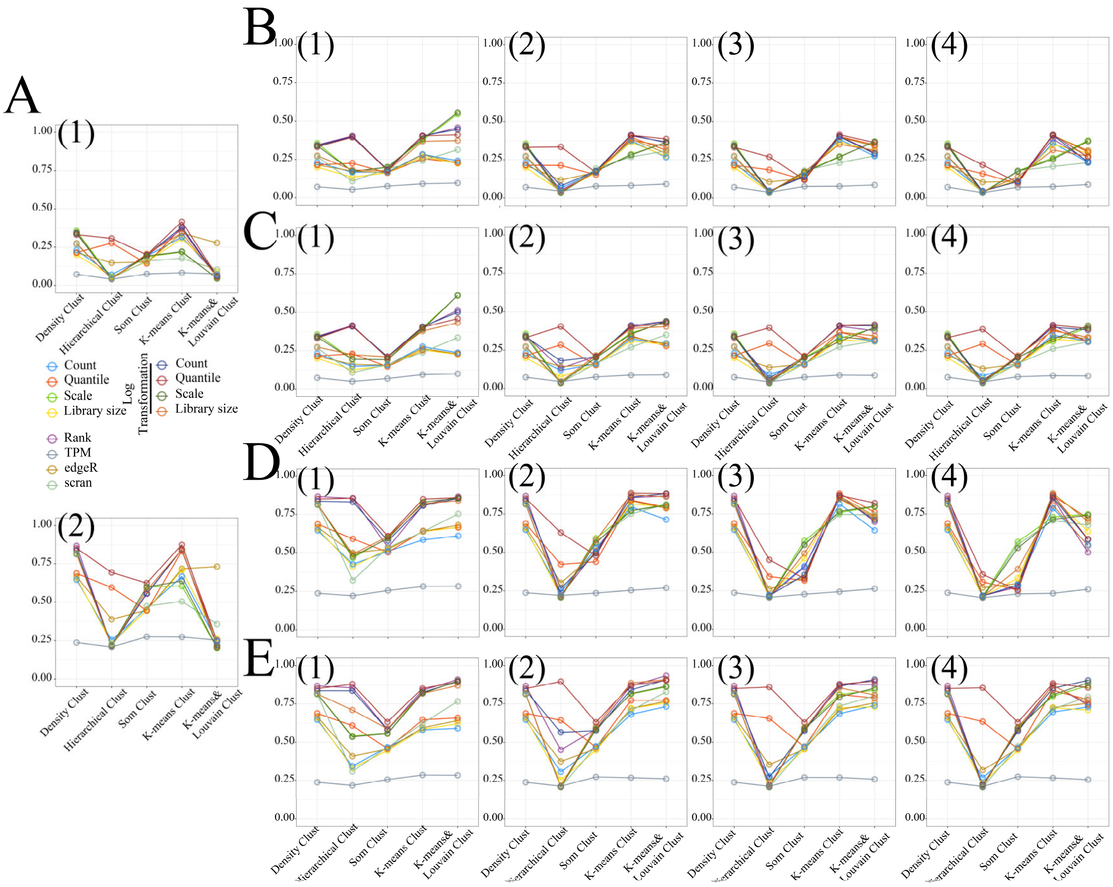

Figure S4. Classification accuracy of different clustering methods under different dimension reduction and normalization methods. (**A**) Classification accuracy without gene feature dimension reduction. (1. Accuracy for sub-category; 2. accuracy for major-category) (**B**) Sub-category classification accuracy with ICA reduction. (**C**) Sub-category classification accuracy with PCA reduction. (**D**) Major-category classification accuracy with ICA reduction. (**E**) Major-category classification accuracy with PCA reduction. (the column of picture labelled by (1): 10 features; the column of picture labelled by (2): 40 features; the column of picture labelled by (3): 70 features; the column of picture labelled by (4): 100 features)

Table S1. The information of real data sequencing files.

First batch of sequencing data					
**File name**	**Sequencing library platform**	**File type**	**Sample**	**File size**	**Read number**
DA1_R1_001.fastq	10 × genomics	transcriptome paired-end seq (Read 1)	DA1	132.69G	386680804
DA1_R2_001.fastq	10 × genomics	transcriptome paired-end seq (Read 2)	DA1	132.69G	386680804
DA2_R1_001.fastq	10 × genomics	transcriptome paired-end seq (Read 1)	DA2	140.51 G	420884102
DA2_R2_001.fastq	10 × genomics	transcriptome paired-end seq (Read 2)	DA2	140.51G	420884102
DA3_R1_001.fastq	10 × genomics	transcriptome paired-end seq (Read 1)	DA3	124.12G	361699727
DA3_R2_001.fastq	10 × genomics	transcriptome paired-end seq (Read 2)	DA3	124.12G	361699727

DA4_R1_001.fastq	10 × genomics	transcriptome paired-end seq (Read 1)	DA4	132.40G	395492415
DA4_R2_001.fastq	10 × genomics	transcriptome paired-end seq (Read 2)	DA4	132.40G	395492415
DA5_R1_001.fastq	10 × genomics	transcriptome paired-end seq (Read 1)	DA5	100.12G	291650611
DA5_R2_001.fastq	10 × genomics	transcriptome paired-end seq (Read 2)	DA5	100.12G	291650611
**Second batch of sequencing data**					
**File name**	**Sequencing library platform**	**File type**	**Sample**	**File size**	**Read number**
DA1_R1_002.fq	10 × genomics	transcriptome paired-end seq (Read 1)	DA1	334.98G	976141529
DA1_R2_002.fq	10 × genomics	transcriptome paired-end seq (Read 2)	DA1	334.98G	976141529
DA2_R1_002.fq	10 × genomics	transcriptome paired-end seq (Read 1)	DA2	294.8G	883036589
DA2_R2_002.fq	10 × genomics	transcriptome paired-end seq (Read 2)	DA2	294.8G	883036589
DA3_R1_002.fq	10 × genomics	transcriptome paired-end seq (Read 1)	DA3	309.24G	901133794
DA3_R2_002.fq	10 × genomics	transcriptome paired-end seq (Read 2)	DA3	309.24G	901133794
DA4_R1_002.fq	10 × genomics	transcriptome paired-end seq (Read 1)	DA4	294.13G	857103666
DA4_R2_002.fq	10 × genomics	transcriptome paired-end seq (Read 2)	DA4	294.13G	857103666
DA5_R1_002.fq	10 × genomics	transcriptome paired-end seq (Read 1)	DA5	99.52G	298088853
DA5_R2_002.fq	10 × genomics	transcriptome paired-end seq (Read 2)	DA5	99.52G	298088853

Table S2. The information of read data re-sequencing simulation data.

**Simulation data name**	**Fragment type**	**Sequencing type**	**File size**	**Read number（ten thousand）**
simulation_sequence_R1.fq	tag-based fragment	paired-end	87.92 G	30000
simulation_sequence_R2.fq			87.91 G	30000

Table S3. the information of collected data from GPL96 platform for augmentation-formed ground truth.

Major-categories	Sample ID	Sample description	Geo number	Sub-categories
B cells	GSM565308	B.cells.naive	GSE22886	Naive B cells
	GSM565309	B.cells.naive	GSE22886	
	GSM565310	B.cells.naive	GSE22886	
	GSM565311	B.cells.naive	GSE22886	
	GSM565312	B.cells.naive	GSE22886	
	GSM565313	B.cells.naive	GSE22886	
	GSM565314	B.cells.naive	GSE22886	
	GSM565315	B.cells.memory	GSE22886	Memory B cells
	GSM565316	B.cells.memory	GSE22886	
	GSM565317	B.cells.memory	GSE22886	
	GSM565318	B.cells.memory	GSE22886	
	GSM565319	B.cells.memory	GSE22886	
	GSM565320	B.cells.memory	GSE22886	
	GSM565321	B.cells.memory	GSE22886	
	GSM565322	B.cells.memory	GSE22886	
	GSM417263	B.germinal.center	GSE16712	Germinal center B cells
	GSM417264	B.germinal.center	GSE16712	
	GSM417265	B.germinal.center	GSE16712	
	GSM417266	B.germinal.center	GSE16712	
	GSM322407	B.Tonsil	GSE12845	Tonsil B cells
	GSM322408	B.Tonsil	GSE12845	
	GSM322409	B.Tonsil	GSE12845	
	GSM1305903	B.Tonsil.CD40.activated	GSE54017	Tonsil activated CD40 B cells
	GSM1305905	B.Tonsil.CD40.activated	GSE54017	
	GSM1305907	B.Tonsil.CD40.activated	GSE54017	
	GSM1305909	B.Tonsil.CD40.activated	GSE54017	
	GSM99339	B.CLL	GSE4392	CLL B cells
	GSM99340	B.CLL	GSE4392	
	GSM99341	B.CLL	GSE4392	
	GSM99347	B.CLL	GSE4392	
	GSM99348	B.CLL	GSE4392	
	GSM99349	B.CLL	GSE4392	
T cells	GSM24511	T.progenitor.cells	GSE1460	Progenitor T cells
	GSM24609	T.progenitor.cells	GSE1460	
	GSM24610	T.progenitor.cells	GSE1460	
	GSM24613	thymocytes	GSE1460	Thymocytes
	GSM24614	thymocytes	GSE1460	
	GSM24615	thymocytes	GSE1460	
	GSM565269	T.cells.CD8	GSE22886	CD8 T cells
	GSM565270	T.cells.CD8	GSE22886	
	GSM565271	T.cells.CD8	GSE22886	
	GSM565272	T.cells.CD8	GSE22886	
	GSM565287	T.cells.CD4.memory.resting	GSE22886	Resting memory CD4 T cells
	GSM565288	T.cells.CD4.memory.resting	GSE22886	
	GSM565289	T.cells.CD4.memory.resting	GSE22886	
	GSM565290	T.cells.CD4.memory.activated	GSE22886	Activated memory CD4 T cells
	GSM565291	T.cells.CD4.memory.activated	GSE22886	
	GSM565292	T.cells.CD4.memory.activated	GSE22886	
	GSM607540	Tregs	GSE24634	Tregs
	GSM607541	Tregs	GSE24634	
	GSM607542	Tregs	GSE24634	
	GSM607546	Teffs	GSE24634	Teffs
	GSM607547	Teffs	GSE24634	
	GSM607548	Teffs	GSE24634	
	GSM135264	T.prolymphocytic.leukemia	GSE5788	Leukemia prolymphocytic T cells

	GSM135265	T.prolymphocytic.leukemia	GSE5788	
	GSM135266	T.prolymphocytic.leukemia	GSE5788	
	GSM135267	T.prolymphocytic.leukemia	GSE5788	
	GSM135268	T.prolymphocytic.leukemia	GSE5788	
NK cells	GSM565293	NK.cells.resting	GSE22886	Resting NK cells
	GSM565294	NK.cells.resting	GSE22886	
	GSM565295	NK.cells.resting	GSE22886	
	GSM565296	NK.cells.resting	GSE22886	
	GSM565297	NK.cells.activated	GSE22886	Activated NK cells
	GSM565298	NK.cells.activated	GSE22886	
	GSM565299	NK.cells.activated	GSE22886	
	GSM565300	NK.cells.activated	GSE22886	
	GSM565301	NK.cells.activated	GSE22886	
	GSM565302	NK.cells.activated	GSE22886	
	GSM565303	NK.cells.activated	GSE22886	
	GSM565304	NK.cells.activated	GSE22886	
	GSM565305	NK.cells.activated	GSE22886	
	GSM565306	NK.cells.activated	GSE22886	
	GSM565307	NK.cells.activated	GSE22886	
Monocytes to Macrophages	GSM565330	Monocytes	GSE22886	Monocytes
	GSM565331	Monocytes	GSE22886	
	GSM565332	Monocytes	GSE22886	
	GSM565333	Monocytes	GSE22886	
	GSM565334	Monocytes	GSE22886	
	GSM565335	Monocytes	GSE22886	
	GSM565336	Monocytes	GSE22886	
	GSM565337	Monocytes	GSE22886	
	GSM565338	Monocytes	GSE22886	
	GSM565339	Monocytes	GSE22886	
	GSM565340	Monocytes	GSE22886	
	GSM565341	Monocytes	GSE22886	
	GSM205591	Monocytes.16	GSE8286	Monocytes 16h
	GSM205592	Monocytes.16	GSE8286	
	GSM205594	Monocytes.16	GSE8286	
	GSM1340127	Monocytes.activated	GSE55608	Activated Monocytes
	GSM1340128	Monocytes.activated	GSE55608	
	GSM1340129	Monocytes.activated	GSE55608	
	GSM565354	Macrophages.M0	GSE22886	Macrophages M0
	GSM565355	Macrophages.M0	GSE22886	
	GSM565356	Macrophages.M0	GSE22886	
	GSM565357	Macrophages.M0	GSE22886	
	GSM565358	Macrophages.M0	GSE22886	
	GSM565359	Macrophages.M0	GSE22886	
	GSM565360	Macrophages.M0	GSE22886	
	GSM565361	Macrophages.M0	GSE22886	
	GSM565362	Macrophages.M0	GSE22886	
	GSM565363	Macrophages.M0	GSE22886	
	GSM565364	Macrophages.M0	GSE22886	
	GSM565365	Macrophages.M0	GSE22886	
	GSM115055	Macrophages.M1	GSE5099	Macrophages M1
	GSM115056	Macrophages.M1	GSE5099	
	GSM115057	Macrophages.M1	GSE5099	
	GSM115058	Macrophages.M2	GSE5099	Macrophages M2
	GSM115059	Macrophages.M2	GSE5099	
	GSM115060	Macrophages.M2	GSE5099	
	GSM267445	macrophages.decidual	GSE10612	Decidual macrophages
	GSM267447	macrophages.decidual	GSE10612	
	GSM267449	macrophages.decidual	GSE10612	
	GSM267451	macrophages.decidual	GSE10612	
	GSM267453	macrophages.decidual	GSE10612	
Dendritic cells	GSM565366	Dendritic.cells.resting	GSE22886	Resting Dendritic cells

	GSM565367	Dendritic.cells.resting	GSE22886	
	GSM565368	Dendritic.cells.resting	GSE22886	
	GSM565369	Dendritic.cells.resting	GSE22886	
	GSM565370	Dendritic.cells.resting	GSE22886	
	GSM565371	Dendritic.cells.resting	GSE22886	
	GSM565372	Dendritic.cells.activated	GSE22886	Activated Dendritic cells
	GSM565373	Dendritic.cells.activated	GSE22886	
	GSM565374	Dendritic.cells.activated	GSE22886	
	GSM565375	Dendritic.cells.activated	GSE22886	
	GSM565376	Dendritic.cells.activated	GSE22886	
	GSM565377	Dendritic.cells.activated	GSE22886	
	GSM1141025	mDC	GSE46913	mDC
	GSM1141026	mDC	GSE46913	
	GSM1141027	mDC	GSE46913	
	GSM1141028	mDC	GSE46913	
	GSM1141029	mDC	GSE46913	
	GSM1141074	mDC	GSE46913	
	GSM1141075	mDC	GSE46913	
	GSM1141076	mDC	GSE46913	
	GSM1141077	mDC	GSE46913	
	GSM1141078	mDC	GSE46913	
Plasma cells	GSM565323	Plasma.cells.1	GSE22886	Plasma cells 1
	GSM565324	Plasma.cells.1	GSE22886	
	GSM565325	Plasma.cells.1	GSE22886	
	GSM565326	Plasma.cells.2	GSE22886	Plasma cells 2
	GSM565327	Plasma.cells.2	GSE22886	
	GSM565328	Plasma.cells.2	GSE22886	
	GSM565329	Plasma.cells.2	GSE22886	
	GSM38155	Plasma.cells.MGUS	GSE2113	Plasma cells MGUS
	GSM38156	Plasma.cells.MGUS	GSE2113	
	GSM38157	Plasma.cells.MGUS	GSE2113	
	GSM38158	Plasma.cells.MGUS	GSE2113	
	GSM38159	Plasma.cells.MGUS	GSE2113	
	GSM38160	Plasma.cells.MGUS	GSE2113	
	GSM38161	Plasma.cells.MGUS	GSE2113	
	GSM38201	Plasma.cells.PCL	GSE2113	Plasma cells PCL
	GSM38202	Plasma.cells.PCL	GSE2113	
	GSM38203	Plasma.cells.PCL	GSE2113	
	GSM38204	Plasma.cells.PCL	GSE2113	
	GSM38205	Plasma.cells.PCL	GSE2113	
	GSM38206	Plasma.cells.PCL	GSE2113	
	GSM38162	Plasma.cells.MM	GSE2113	Plasma cells MM
	GSM38163	Plasma.cells.MM	GSE2113	
	GSM38164	Plasma.cells.MM	GSE2113	
	GSM38165	Plasma.cells.MM	GSE2113	
	GSM38166	Plasma.cells.MM	GSE2113	
	GSM38167	Plasma.cells.MM	GSE2113	
CD34 cells	GSM595118	CD34.progenitors.cells.PB	GSE24193	CD34 progenitors cells 1
	GSM595120	CD34.progenitors.cells.PB	GSE24193	
	GSM595122	CD34.progenitors.cells.PB	GSE24193	
	GSM595124	CD34.progenitors.cells.PB	GSE24193	
	GSM76926	CD34.progenitors.cells.PB.IM	GSE3410	CD34 progenitors cells 1IM
	GSM76927	CD34.progenitors.cells.PB.IM	GSE3410	
	GSM76928	CD34.progenitors.cells.PB.IM	GSE3410	
	GSM247172	CD34.progenitors.cells.BM	GSE9827	CD34 progenitors cells 2
	GSM247191	CD34.progenitors.cells.BM	GSE9827	
	GSM247944	CD34.progenitors.cells.BM	GSE9827	
	GSM247958	CD34.progenitors.cells.BM	GSE9827	

	GSM247959	CD34.progenitors.cells.BM.ET	GSE9827	CD34 progenitors cells 2ET
	GSM247962	CD34.progenitors.cells.BM.ET	GSE9827	
	GSM248011	CD34.progenitors.cells.BM.ET	GSE9827	
	GSM248113	CD34.progenitors.cells.BM.ET	GSE9827	
	GSM248157	CD34.progenitors.cells.BM.ET	GSE9827	
	GSM248159	CD34.progenitors.cells.BM.ET	GSE9827	
	GSM248161	CD34.progenitors.cells.BM.ET	GSE9827	
	GSM248164	CD34.progenitors.cells.BM.ET	GSE9827	
	GSM730669	CD34.progenitors.cells.cord	GSE29522	CD34 progenitors cells 3
	GSM730670	CD34.progenitors.cells.cord	GSE29522	
	GSM730671	CD34.progenitors.cells.cord	GSE29522	
	GSM730672	CD34.progenitors.cells.cord	GSE29522	
	GSM730673	CD34.progenitors.cells.cord	GSE29522	
	GSM730674	CD34.progenitors.cells.cord	GSE29522	
	GSM730675	CD34.progenitors.cells.cord	GSE29522	
	GSM730676	CD34.progenitors.cells.cord	GSE29522	
Neutrophils	GSM565378	Neutrophils	GSE22886	Neutrophils
	GSM565379	Neutrophils	GSE22886	
	GSM565380	Neutrophils	GSE22886	
	GSM565381	Neutrophils	GSE22886	
	GSM565382	Neutrophils	GSE22886	
	GSM66867	Neutrophils.Patient	GSE3037	Neutrophils patient
	GSM66868	Neutrophils.Patient	GSE3037	
	GSM66869	Neutrophils.Patient	GSE3037	
	GSM66870	Neutrophils.Patient	GSE3037	
	GSM66871	Neutrophils.Patient	GSE3037	
	GSM66872	Neutrophils.Patient	GSE3037	
	GSM66873	Neutrophils.Patient	GSE3037	
	GSM66874	Neutrophils.Patient	GSE3037	
Erythroblast	GSM266842	Erythroblast	GSE10584	Erythroblast
	GSM266844	Erythroblast	GSE10584	
	GSM266902	Erythroblast	GSE10584	
	GSM266910	Erythroblast	GSE10584	
	GSM266926	Erythroblast	GSE10584	
	GSM266927	Erythroblast	GSE10584	
	GSM266848	Erythroblast.InLu	GSE10584	Erythroblast InLu
	GSM266850	Erythroblast.InLu	GSE10584	
	GSM266904	Erythroblast.InLu	GSE10584	
	GSM266924	Erythroblast.InLu	GSE10584	
	GSM266929	Erythroblast.InLu	GSE10584	
	GSM266930	Erythroblast.InLu	GSE10584	
Endothelial	GSM356585	Endothelial.cells.HUVEC	GSE14230	Endothelial cells HUVEC
	GSM356586	Endothelial.cells.HUVEC	GSE14230	
	GSM356587	Endothelial.cells.HUVEC	GSE14230	
	GSM356588	Endothelial.cells.HUVEC	GSE14230	
	GSM356589	Endothelial.cells.HUVEC	GSE14230	
	GSM356590	Endothelial.cells.BM.P	GSE14230	Endothelial cells P
	GSM356591	Endothelial.cells.BM.P	GSE14230	
	GSM356592	Endothelial.cells.BM.P	GSE14230	
	GSM356593	Endothelial.cells.BM.P	GSE14230	
	GSM356594	Endothelial.cells.BM.P	GSE14230	

Table S4. The information of simulation data files of augmentation-formed ground truth.

**Main-gradient for augmentation-formed ground truth**				
**simulation data name**	**fragment type**	**sequencing type**	**file size**	**read number（ten thousand）**
RDc.1_R1.fq	tag-based fragment	paired-end	3 MB	10
RDc.1_R2.fq			3 MB	10
RDc.2_R1.fq	tag-based fragment	paired-end	299.85 MB	100
RDc.2_R2.fq			299.81 MB	100
RDc.3_R1.fq	tag-based fragment	paired-end	2.93 G	1000
RDc.3_R2.fq			2.93 G	1000
RDc.4_R1.fq	tag-based fragment	paired-end	29.31 G	10000
RDc.4_R2.fq			29.30 G	10000
**Sub-gradient for augmentation-formed ground truth**				
**simulation data name**	**fragment type**	**sequencing type**	**file size**	**read number（ten thousand）**
RDc.2.1_R1.fq	tag-based fragment	paired-end	600.03 MB	200
RDc.2.1_R2.fq			599.88 MB	200
RDc.2.2_R1.fq	tag-based fragment	paired-end	900.06 MB	300
RDc.2.2_R2.fq			899.82 MB	300
RDc.2.3_R1.fq	tag-based fragment	paired-end	1.17 G	400
RDc.2.3_R2.fq			1.17 G	400
RDc.2.4_R1.fq	tag-based fragment	paired-end	1.46 G	500
RDc.2.4_R2.fq			1.46 G	500
RDc.2.5_R1.fq	tag-based fragment	paired-end	1.76 G	600
RDc.2.5_R2.fq			1.76 G	600
RDc.2.6_R1.fq	tag-based fragment	paired-end	2.05 G	700
RDc.2.6_R2.fq			2.05 G	700
RDc.2.7_R1.fq	tag-based fragment	paired-end	2.34 G	800
RDc.2.7_R2.fq			2.34 G	800
RDc.2.8_R1.fq	tag-based fragment	paired-end	2.64 G	900
RDc.2.8_R2.fq			2.64 G	900

Table S5. The information of simulation data files analogous to real data.

**simulation data name**	**fragment type**	**sequencing type**	**file size**	**read number（ten thousand）**
RDd.1_R1.fq	tag-based fragment	paired-end	749.95 MB	250
RDd.1_R2.fq			749.76 MB	250
RDd.2_R1.fq	tag-based fragment	paired-end	748.74 MB	250
RDd.2_R2.fq			748.54 MB	250
RDd.3_R1.fq	tag-based fragment	paired-end	750.08 MB	250
RDd.3_R2.fq			749.88 MB	250
RDd.4_R1.fq	tag-based fragment	paired-end	748.58 MB	250
RDd.4_R2.fq			748.38 MB	250
RDd.5_R1.fq	tag-based fragment	paired-end	749.92 MB	250
RDd.5_R2.fq			749.71 MB	250
RDd.6_R1.fq	tag-based fragment	paired-end	748.73 MB	250
RDd.6_R2.fq			748.53 MB	250
RDd.7_R1.fq	tag-based fragment	paired-end	748.64 MB	250
RDd.7_R2.fq			748.44 MB	250
RDd.8_R1.fq	tag-based fragment	paired-end	748.65 MB	250
RDd.8_R2.fq			748.46 MB	250

RDd.9_R1.fq	tag-based fragment	paired-end	749.00 MB	250
RDd.9_R2.fq			748.80 MB	250
RDd.10_R1.fq	tag-based fragment	paired-end	749.09 MB	250
RDd.10_R2.fq			748.88 MB	250
